# Multispectral imaging flow cytometry reveals distinct frequencies of γ-H2AX foci induction in DNA double strand break repair defective human cell lines

**DOI:** 10.1002/cyto.a.21171

**Published:** 2012-02

**Authors:** Emma C Bourton, Piers N Plowman, Sheba Adam Zahir, Gonul Ulus Senguloglu, Hiba Serrai, Graham Bottley, Christopher N Parris

**Affiliations:** 1 Brunel Institute of Cancer Genetics and Pharmacogenomics, Division of Biosciences, School of Health Sciences and Social Care, Brunel UniversityUxbridge, Middlesex UB8 3PH, United Kingdom; 2 Department of Radiotherapy, St Bartholomew's HospitalWest Smithfield, London EC1A 7BE, United Kingdom; 3 Cronus Technologies Ltd.4 Camberley Business Centre, Camberley, Surrey GU15 3DP

**Keywords:** imaging flow cytometry, DNA double strand break repair, γ-H2AX foci, ionising radiation

## Abstract

The measurement of γ-H2AX foci induction in cells provides a sensitive and reliable method for the quantitation of DNA damage responses in a variety of cell types. Accurate and rapid methods to conduct such observations are desirable. In this study, we have employed the novel technique of multispectral imaging flow cytometry to compare the induction and repair of γ-H2AX foci in three human cell types with different capacities for the repair of DNA double strand breaks (DSB). A repair normal fibroblast cell line MRC5-SV1, a DSB repair defective ataxia telangiectasia (AT5BIVA) cell line, and a DNA-PKcs deficient cell line XP14BRneo17 were exposed to 2 Gy gamma radiation from a ^60^Cobalt source. Thirty minutes following exposure, we observed a dramatic induction of foci in the nuclei of these cells. After 24 hrs, there was a predictable reduction on the number of foci in the MRC5-SV1 cells, consistent with the repair of DNA DSB. In the AT5BIVA cells, persistence of the foci over a 24-hr period was due to the failure in the repair of DNA DSB. However, in the DNA-PKcs defective cells (XP14BRneo17), we observed an intermediate retention of foci in the nuclei indicative of partial repair of DNA DSB. In summary, the application of imaging flow cytometry has permitted an evaluation of foci in a large number of cells (20,000) for each cell line at each time point. This provides a novel method to determine differences in repair kinetics between different cell types. We propose that imaging flow cytometry provides an alternative platform for accurate automated high through-put analysis of foci induction in a variety of cell types. © 2011 International Society for Advancement of Cytometry

DNA double strand breaks (DSB) in eukaryotic cells are potentially lethal lesions and must be rapidly repaired in order to ensure cell survival and maintain genomic integrity (1). DNA DSB can occur via a variety of routes including exposure to exogenous ionising radiation during clinical anticancer radiotherapy (2), exposure to certain classes of anticancer chemotherapeutic drugs (3), and as a result of endogenously derived reactive oxygen species as a consequence of normal cellular metabolism (1).

A critical event in the signaling and subsequent repair of DSB is the phosphorylation of the minor histone protein variant H2AX by the product of the ataxia telangiectasia mutated gene (*ATM*) to form the γ-H2AX protein. Many thousands of γ-H2AX molecules accumulate at the sites of DSB to form nuclear foci where the number of foci is indicative of DNA DSB (4). The phosphorylation of H2AX to form γ-H2AX is an important initial step in the repair of DNA DSB, and under normal cellular physiological conditions, repair of the DSB will occur in a predictable manner (5). In mammalian cells, two distinct biochemical pathways are responsible for repairing DSB: homologous recombination (HR) and nonhomologous end joining (NHEJ) (1). NHEJ is the predominant repair pathway whereby the DNA-dependent protein kinase heterodimer containing the Ku80/Ku70 complex binds to DNA ends and recruits DNA-dependent protein kinase catalytic subunit (DNA-PKcs) and its substrate, the Artemis nuclease, which prepares DNA ends for ligation. Ligation is mediated by a protein complex containing DNA ligase IV stabilized and stimulated by XRCC4 and Cernunos-XLF (6). Animal models demonstrate that defects in any component of NHEJ can lead to hypersensitivity to ionising radiation (IR), genome instability, immunodeficiency, and cancer (7). In humans, defects in the specific genetic components of NHEJ also result in predisposition to lymphoma and leukemia or to extreme radiosensitivity during radiotherapy (8, 9). Alternatively in cells traversing the cell cycle (specifically in S and G2 phase), DSB repair is mediated primarily by the homologous recombination (HR) pathway in an error free manner (1). HR repair is mediated through the Rad50 epistasis group of proteins which bind to the ends of the DSB to protect from exonuclease destruction. A Holliday junction is created whereby lost genetic information is “copied” from the homologous sister chromatid. Following resolution and separation of the two sister chromatids, DNA replication is completed and genomic integrity at the site of the DSB is maintained (1).

In mammalian cells, the induction of γ-H2AX foci occurs rapidly following DSB formation. However, within 1–3 hrs the majority of DSB are repaired which is associated with the dephosphorylation and disappearance of the nuclear γ-H2AX foci (4). In cells with defective DSB repair, there is a persistence of γ-H2AX foci due to the failure of efficient DNA DSB repair. A classical example in which such a phenotype is observed is in cells derived from patients with the inherited disorder ataxia telangiectasia (A-T). An inactivating mutation in the *ATM* gene which functions in the control of cell cycle arrest and induction of DNA DSB repair results in the persistence of γ-H2AX foci in the nucleus of cells exposed to IR (10). In addition, we have recently demonstrated that in a cell line derived from a cancer patient exhibiting clinical and cellular radiation hypersensitivity, the prolonged appearance of γ-H2AX foci in the nuclei of irradiated cells was due to a defect in the *DNA-PKcs* gene which is a critical component of the NHEJ DSB repair pathway (11).

The persistence of γ-H2AX foci in cells hypersensitive to DNA damaging agents has prompted extensive research into the application of γ-H2AX as a biomarker to predict both tumor response and acute and delayed side effects in cancer patients receiving clinical radiotherapy and/or chemotherapy (2). While there are many factors which may govern tumor and patient response to therapy, some evidence exists that γ-H2AX may be a useful predictor of acute and late radiotherapy induced side-effect in cancer patients. Bourton et al., 2011 (2) have recently demonstrated using nonimaging flow cytometry, that in peripheral blood lymphocytes (PBL) derived from radiotherapy patients that experienced severe acute and delayed normal tissue toxicity, there was a persistence of γ-H2AX foci following exposure to 2 Gy gamma radiation. While a number of similar studies have not demonstrated such a strong correlation between γ-H2AX foci retention and severe normal tissue toxicity (*e.g.*, 12), the relationship between foci persistence and defective DSB repair is well established. Such observations suggest that the application of γ-H2AX or other DNA repair biomarkers might be a useful “diagnostic” test to predict the response of cancer patient to radiotherapy and/or chemotherapy, leading to the development of “individualized therapy” for each patient. There are a growing number of automated platforms for the estimation of γ-H2AX foci in cells following exposure to a variety of DNA damaging agents. *In situ* immunocytochemistry provides an accurate but time consuming method (*e.g.*, 11). Zero resolution flow cytometry offers a more rapid technique allowing the determination of overall fluorescence in many thousands of cells but lacks the ability to quantify the number of foci within each cell (13). However, a development of automated single cell microscopy analysis and laser scanning image analysis provides high through-put and accurate foci estimation (14–17).

In this study, we have employed the technique of imaging flow cytometry using the Imagestream^X^ (Amnis Inc., Seattle, WA) to estimate the number of foci induced in the nuclei of normal and DNA DSB repair defective immortalized human fibroblasts exposed to 2 Gy gamma radiation. By counting and imaging more than 20,000 cells, we were able to demonstrate that in two DSB repair defective cell lines (XP14BRneo17 and AT5BIVA), there was a persistence of γ-H2AX foci in the nuclei of cells for up to 24 hrs postirradiation. However, in a repair normal cell line (MRC5-SV1), rapid induction of foci was followed by DNA repair and disappearance of foci within 3 hrs postirradiation. Interestingly, we observed that the number of foci induced was significantly higher in the DSB repair normal cell line compared with the two repair defective cell strains. We suggest that the application of imaging flow cytometry provides an accurate assessment of the levels of DNA damage in cells and adds to the growing number of high through-put methods for the analysis of DNA damage in cells. Further studies with a wider range of cell types and lineages are required to determine the application of imaging flow cytometry for both scientific research and clinical diagnostic application.

## Materials and Methods

### Cell Culture

The XP14BRneo17 (SV40 immortalized) fibroblast cell line was derived from a human subject defective in the NHEJ pathway and has been described in full elsewhere (11). The AT5BIVA SV40 immortalized fibroblasts are derived from an A-T patient as described by Murnane et al., 1985 (18). The MRC5-SV1 SV40 immortalized lung fibroblast cell line is derived from a repair normal individual (19). Cells were routinely cultured in Dulbecco's Modified Eagle Medium (DMEM) (PAA Laboratories Ltd., Yeovil, Somerset, UK), supplemented with 10% fetal calf serum (PAA Laboratories Ltd.), 2 mM l-glutamine and 100 Units/ml penicillin and streptomycin (PAA Laboratories Ltd.). Cells were grown as monolayers at 37°C in a humidified atmosphere of 5% CO_2_ in air.

### Gamma Radiation Dose Response Curve

To determine the level of fluorescence induced by different doses of gamma radiation, and thus the level of γ-H2AX induction and DNA damage, each of the three cell lines was irradiated as proliferating monolayers with 0, 2, 4, 6, and 8 Gy gamma radiation from a ^60^Cobalt source (Puridec Irradiation Technologies Ltd., Oxon, UK) at a distance of 25 cm from the gamma source which provided a dose rate of 1.3–1.4 Gy/min. On the basis of the dose response curves in each cell line, all future experiments were conducted following a dose of 2 Gy gamma radiation.

### Gamma Irradiation of Cells

To induce DNA DSB and subsequent γ-H2AX foci induction cells growing as proliferating monolayers were irradiated with 2 Gy gamma. For the gamma radiation dose response, cells were fixed at 30 min postirradiation and subjected to Imagestream^X^ analysis to determine total cellular or nuclear fluorescence as described below.

### Cell Fixation and Antibodies Staining

Untreated control cells and cells irradiated with 2 Gy gamma radiation were fixed at 30 min, 3, 5, and 24 hrs postirradiation. Cells were detached by trypsinisation in ∼ 1 ml of 0.25% trypsin-EDTA (Fisher Scientific, Loughborough, Leicester, UK) and washed twice in phosphate buffered saline (PBS) before fixation in 50:50 (V:V) methanol:acetone at 4°C. Cells were stored in fixative for not more than 72 hrs before γ-H2AX antibody staining.

### Antibodies and Immunocytochemistry

Fixed cells were rehydrated with three washes in PBS and then permeabilized in PBS containing 0.25% Triton X-100 (Sigma-Aldrich, Dorset, UK) for 5 min at room temperature (RT). After blocking the cells for 1 hr at RT in blocking buffer comprised of PBS containing 0.1% Triton X-100 and 0.5% nonfat milk protein (Premier International Food Ltd. UK, Spalding, Lincolnshire, UK), cells were incubated at 4°C overnight in mouse monoclonal antiserine^139^ γ-H2AX antibody, clone JBW 301 (Millipore UK Ltd., Hampshire, UK) at 1:10,000 dilution. Following three washes in wash buffer (PBS + 0.1% Triton X-100), cells were incubated for 1 hr at RT in anti-mouse IgG (whole molecule) R-phycoerythrin conjugated antibody diluted to 1:50 in blocking buffer. Following antibody staining, cells were washed three times with 1 ml changes of wash buffer and once in PBS. One hundred microliters of Accumax flow cytometry buffer (PAA Laboratories Ltd.) was applied containing 5 μM Draq 5 (Biostatus Ltd., Leicestershire, UK). Samples for fluorescence compensation were prepared in which either the secondary antibody (R-phycoerythrin conjugate) or Draq 5 was omitted from the procedure.

### *In Situ* Detection of γ-H2AX Foci

The number of γ-H2AX foci detected using *in situ* fluorescence microscopy was compared with the results generated with imaging flow cytometry. Briefly, the three cell lines were grown to ∼ 70–80% confluence on 13 mm glass coverslips and exposed to 2 Gy gamma radiation as described above. For untreated cells (nonirradiated cells) and at 30 min, 3, 5, and 24 hrs postirradiation, three coverslips were fixed in methanol:acetone and antibodies were applied as described. Using an Axioscope 2 fluorescence microscope with a 100-fold magnification objective (Zeiss, Goettingen, Germany), γ-H2AX foci were counted in the nuclei of at least 100 cells for each cell line in untreated cells and those irradiated with 2 Gy gamma radiation at 30 min, 3, 5, and 24 hrs postirradiation.

### Imaging Flow Cytometry

Imaging flow cytometry was conducted using the Imagestream^X^ system (Amnis Inc., Seattle, Washington). This permits image capture of each cell in flow using a maximum of six optical channels. Using the Inspire™ data acquisition software, images of 20,000 cells were captured on channel 1 for brightfield (BF); on channel 3 for phycoerythrin (PE) representing red staining of γ-H2AX staining; and on channel 5 for Draq 5 staining of the nuclear region of each cell. Cell classifiers were applied to the BF channel to capture objects that ranged between 50 and 300 units on an arbitrary scale. These values were determined from previous analyses whereby this classifier range was observed to capture primarily single cell images. Following excitation with a 488 nm laser at a power setting of 75 mW, all images were captured using a 40× objective. Images of cells were acquired at a rate of ∼ 150–200 cell images per second.

### Image Compensation

Image compensation was performed on populations of cells that had been fixed 30 min after exposure to 2 Gy gamma radiation in which PE staining intensity was likely to be highest. Cells that were stained with anti γ-H2AX-PE only or Draq 5 only were used for generating the compensation matrix taking care that images were collected without BF illumination since it was important to capture fluorescence intensity with a single source of illumination (75 mW 488 nm laser). The Ideas™ compensation wizard generates a table of coefficients whereby detected light that is displayed by each image is placed into the proper channel (channel 3 for PE and channel 5 for Draq 5) on a pixel-by-pixel basis. The coefficients are normalized to 1 and each coefficient represents the leakage of fluorochrome into juxtaposed channels. A typical compensation table in a 6 × 6 table format is shown in Supporting Information Table 1. Calculated compensation values were applied to all subsequent analyses as appropriate.

### Analysis of Cell Images and Calculation of Foci Number

The raw image file (.rif) of the cells exposed to 2 Gy gamma radiation at 30 min postexposure was loaded into the Ideas™ application software. This file was chosen first due to the likely highest induction of γ-H2AX foci and brightest intensity of PE. Images of each cell in channel 1 brightfield (BF), channel 3 PE intensity (γ-H2AX fluorescence), and channel 5 Draq 5 (nuclear morphology) were analyzed using Ideas™. The image gallery properties were adjusted to optimize the displayed fluorescent images.

The analysis process comprises a multistep process and initially uses a series of predefined “building blocks” provided within the software. These tools generate a series of scatter and histogram plots which allow the identification of single and focused cells, stained for both Draq 5 (nucleus) and PE (γ-H2AX foci). Identification of single cells is determined by the creation of a scatter plot of the population defined by cell area and cell aspect ratio from the BF image, whereby each dot within the scatter represents a single cell. The scatterplot single cell region was defined by visually validating the bright-field images, and finally gated using the polygon region tool (Supporting Information Fig. 1A).

**Figure 1:**
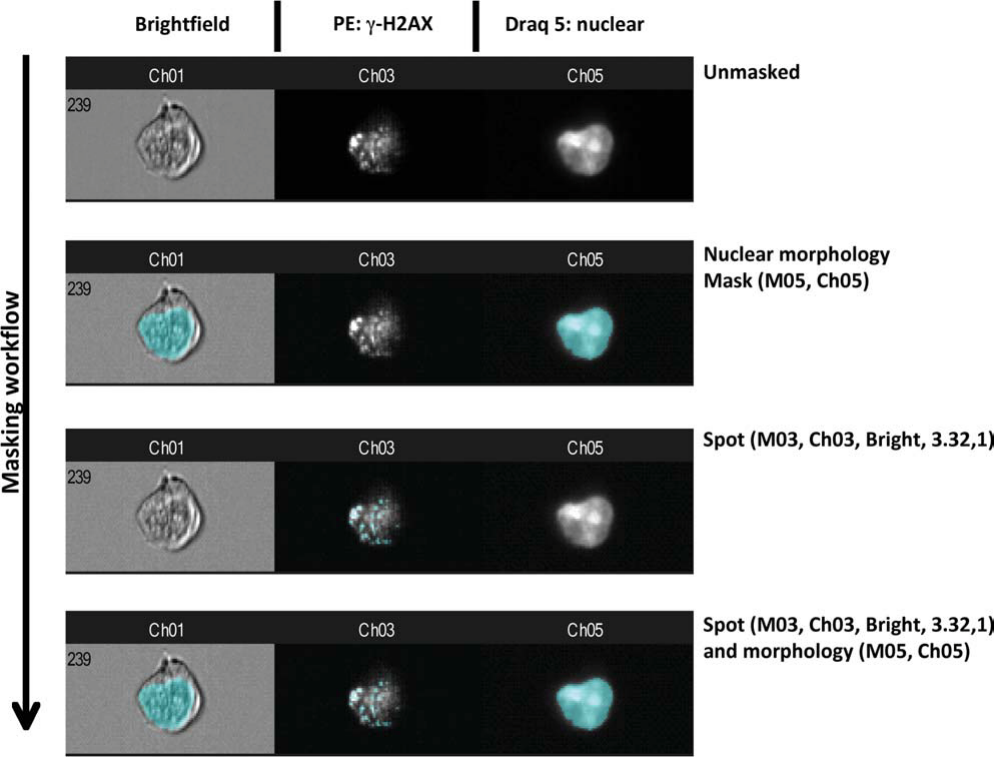
The strategy whereby nuclear γ-H2AX foci were identified, masked, and enumerated is shown. Here, an example of a cell in brightfield, channel 3 (γ-H2AX foci), and channel 5 (nuclear staining by Draq 5) is shown in an unmasked format. Next a nuclear morphology mark is applied after which a spot mask is applied to channel 3 showing γ-H2AX foci. Finally, spots (γ-H2AX foci) masking within the nuclear region is shown. The associated name path for foci calculation from the Ideas software is shown to the right of the images.

Using the “In Focus” tool, a histogram of the single cell population gated above was produced. The cells were distributed in histogram “bins” based on the calculated focus of the BF images. Clicking on individual histogram bins allows visualization of all images within that histogram bin in the image gallery. Single cells in focus were then defined by using the line region tool (Supporting Information Fig. 1B). The fluorescence positive two color tool generated a scatter plot of single and focussed cells stained for both γ-H2AX (PE) and Draq 5 (nucleus). The polygon tool was then utilized to gate the cells (Supporting Information Fig. 1C).

### Masking Strategy to Determine the Number of γ-H2AX Foci in Each Nucleus

**Figure 2:**
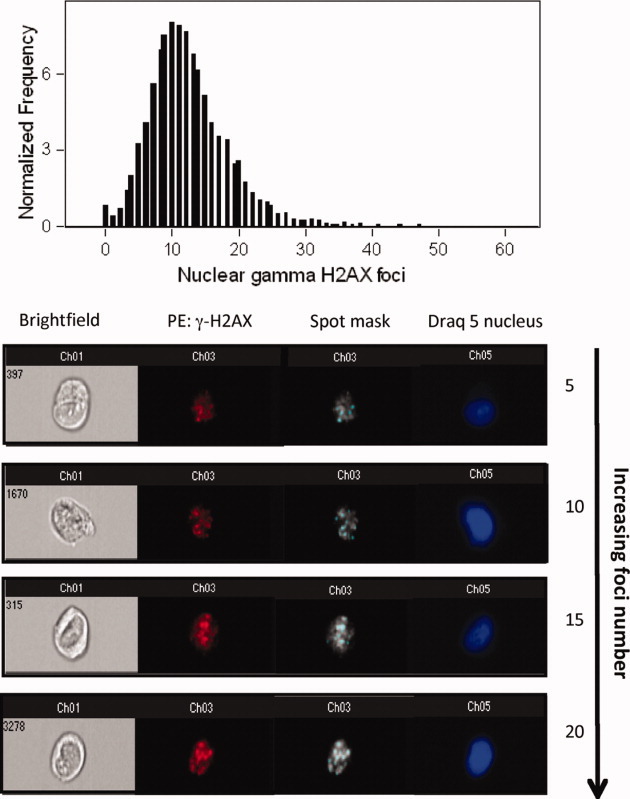
This figure shows a typical distribution plot of γ-H2AX foci in ∼ 20,000 cells following exposure to 2 Gy gamma radiation. Additionally, multispectral images of cells with increasing numbers of γ-H2AX foci within the nucleus are shown. Each panel depicts a brightfield image (channel 1) of the cell followed by an image of γ-H2AX foci within the nucleus with and without the spot mask. To visualize the spot mask, images have been presented in grayscale. Finally, an image of each cell stained with Draq 5 identifying the nucleus is shown.

To determine the number of γ-H2AX foci within the nucleus of each cell type, a series of masks were created which identified the region of interest in a manner similar to that described by Filby et al., 2011 (20). Using the Mask Manager of the Ideas™ software, a nuclear morphology mask was created using the Draq 5 (channel 5) image of each cell. To accurately determine the number of γ-H2AX foci within each cell, a “truth population” was identified using the image gallery whereby images were identified with known number of channel 3 (PE) foci ranging from zero to 30 or more foci. Using this truth population, a spot mask was created using the spot function of the Mask Manager tool. Using spot-to-background ratio, foci (intensity peaks) were masked that were at least twofold greater than the background with a minimum diameter of 2 pixels. Finally to determine the number of foci within the nuclear specific region of each cell, a combined mask was created by utilizing Boolean logic. A masking workflow is shown in Figure 1. Here images are shown in BF (channel 1), PE staining for γ-H2AX foci (in grayscale on channel 3), and Draq 5 staining identifying the nucleus (in grayscale on channel 5). Cells are shown unmasked followed by application of the nuclear mask, then the spot mask for γ-H2AX foci and lastly the localization of spots within the nuclear region is shown. The associated name path from the Ideas™ software for the derivation of the masks is also shown. Finally, using the Features option in the Ideas software, foci in all cells were enumerated and plotted as a histogram of normalized frequency of foci against foci number. An example of foci distribution is shown in Figure 2. Once this analysis procedure had been completed for the 30 min time point postirradiation exposure, the data file was saved as a template which was then applied to the analysis of all other time points (untreated control, 3, 5, and 24 hrs) for each cell line. A separate template file was derived and visually validated specifically for each cell line.

### Experimental Timescale

All data generated in this article was derived over a 1-year timescale between January 2010 and August 2011.

## Results

### Gamma Radiation Dose Response Curve

The induction of fluorescence in the three cell lines was determined by counting 20,000 cells with the Imagestream^X^ following exposure to 0, 2, 4, 6, and 8 Gy gamma radiation. To determine nuclear fluorescence attributable to PE staining (γ-H2AX), a nuclear morphology mask was created using the Mask Manager in Ideas™ after which the intensity of masked PE staining (γ-H2AX) in the nucleus was derived. The dose response curve can be seen in Supporting Information Figure 2. It can be observed that for each increment in dose, there is a 1- to 1.5-fold increase in nuclear fluorescence. For all subsequent experiments, a dose of 2 Gy radiation was used to induce γ-H2AX foci.

### γ-H2AX Foci Quantitation Using the Imagestream^X^

**Figure 3:**
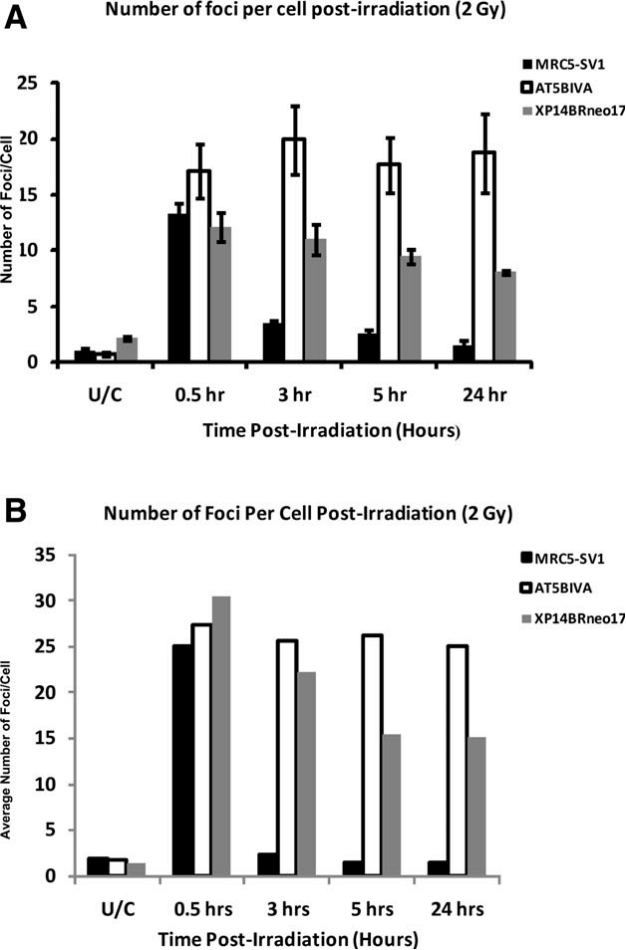
The mean number of γ-H2AX foci after analysis using the Imagestream^X^ in MRC5-SV1, AT5BIVA and XP14BRneo17 cells is shown in Figure 3A. Average foci numbers are shown in untreated control cells and at 30 min, 3, 5, and 24 hrs after exposure to 2 Gy gamma radiations. Error bars represent standard error of the mean derived from three independent experiments in which 20,000 cells at each time point were analyzed. Figure 3B shows a distribution analysis of γ-H2AX foci in the nuclei of the three cell lines in untreated cells and in cells at 30 min, 3, 5, and 24 hrs after exposure to 2 Gy gamma radiation using *in situ* microscopy. Data here are derived from counting at least 100 cells at each time point.

The average number of foci per cell in each of the three cell lines analyzed with the Imagestream^X^ is shown in Figure 3A where the data are derived from three independent experiments. γ-H2AX foci counts were determined by enumerating the number of spots in the spot mask as demonstrated in Figures 1 and 2. In unirradiated cells, the mean number of foci per nucleus is 1.13 for the repair normal MRC5-SV1 fibroblasts and 0.76 for the DSB repair defective AT5BIVA cells. In the DSB repair defective XP14BRneo17 cells, the mean number of spontaneous foci is 2.21 foci per cell. Following exposure of the cells to 2 Gy gamma radiation at a dose rate of 1.3 to 1.4 Gy per min, there is a significant increase in the number of foci which is consistent with the induction of DNA DSB as a consequence of radiation exposure. The MRC5-SV1 repair normal cells exhibit 14.04 foci per cell. In the DNA DSB repair defective AT5BIVA and XP14BRneo17 cells, an average of 17.17 and 12.26 foci per cell, respectively, is observed. During the course of 24 hrs postradiation exposure, the three cell lines behaved differently in terms of the persistence and/or removal of the γ-H2AX foci. After 3 hrs, the repair normal MRC5-SV1 cells resolve the radiation-induced DNA DSB and there is a reduction in the number of foci to near unirradiated levels with 1.58 foci per cell. Consistent with the DSB repair defect in the AT5BIVA cells, there is a persistence of γ-H2AX foci within the nuclei of cells at 24 hrs postirradiation with 18.72 foci per cell. Interestingly, in the XP14BRneo17 cells which are also defective in DNA DSB repair, there is an intermediate level of persistence of γ-H2AX foci with an average of 8.10 foci per cell after 24 hrs. This is significantly different from other cell lines examined in this study and is likely a reflection of a subtle difference in the defect in DNA DSB repair when compared with both the AT5BIVA and MRC5-SV1 normal cells. In Figure 2, images of sample cells with 5, 10, 15, and 20 foci are presented together with the associated spot mask. In this example, cells were fixed at 30 min postirradiation and captured with the Imagestream^X^.

The application of a Student's unpaired *t*-test demonstrates that differences in the persistence of γ-H2AX foci within each cell line are statistically significant with 95% confidence limits. For example, the AT5BIVA cells retain significantly more foci than the repair normal MRC5-SV1 cells after 24 hrs (*P* < 0.008). Similarly, the XP14BRneo17 cells retain significantly more foci the MRC5-SV1 cells at 24 hrs postexposure (*P* < 0.001). Finally, the AT5BIVA cells retain significantly more foci than the XP14BRneo17 cells at 24 hrs (*P* < 0.05).

### *In Situ* Quantitation of γ-H2AX Foci

To validate the γ-H2AX foci counts derived from the Imagestream^X^ analysis, the induction of foci within the three cell lines was also investigated using *in situ* immunofluorescence, and the data is shown in Figure 3B. It can be seen that (using a fluorescence microscope with a 100× magnification objective) the mean number of foci observed in the three cell lines following exposure to 2 Gy gamma radiation is higher than that observed using the Imagestream^X^. However, the relative difference between the three cell types is retained.

### Comparison of Total and Nuclear Fluorescence

**Figure 4:**
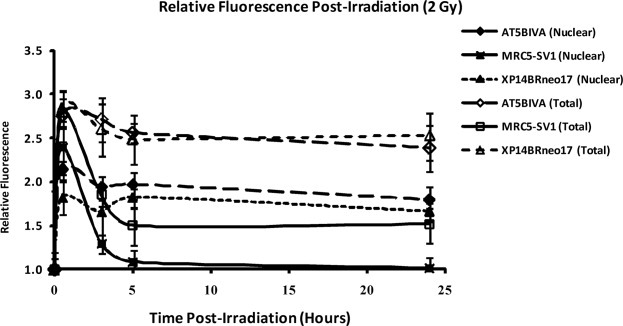
The relative level of nuclear fluorescence (closed symbols) and total cellular fluorescence (open symbols) in the MRC5-SV1, AT5BIVA, and XP14BRneo17 cells is shown in Figure 3. Relative fluorescence is calculated by dividing the level of fluorescence observed in irradiated cells by the fluorescence level in untreated cells. The dramatic increase in fluorescence is indicative of the presence of γ-H2AX foci in the nuclei of the cells. This is followed by rapid repair in the repair normal MRC5-SV1 but retention of fluorescence in the DSB repair defective AT5BIVA and XP14BRneo17 cells. Error bars represent standard error of the mean derived from three independent experiments in which ∼ 20,000 cells at each time point were analyzed.

The overall relative nuclear fluorescence (on channel 3 of the Imagestream^X^) attributable to γ-H2AX foci induction can be readily compared with the total cellular fluorescence (also channel 3). Total cellular fluorescence in this scenario is analogous to classical (zero resolution) flow cytometry and consequently provides a useful comparison between imaging and classical flow. The data shown in Figure 4 compares PE-associated nuclear and total cellular fluorescence before and after gamma radiation exposure in the three cell lines. The data broadly mimics the induction and removal of γ-H2AX foci in the nuclei of irradiated cells. In the repair competent MRC5-SV1 cells, there is a dramatic induction of nuclear fluorescence (γ-H2AX) foci at 30 min post 2 Gy gamma irradiation. However, the relative nuclear fluorescence quickly returns to unirradiated levels which is consistent with the repair of radiation induced DNA DSB. In the AT5BIVA cells, there is persistence of relative fluorescence within the nuclei of the cells due to the failure of DSB repair and the retention of γ-H2AX foci as demonstrated in Figure 3A.

A similar pattern of nuclear fluorescence retention is observed in the XP14BRneo17 cells which are also characterized by a defect in DNA DSB repair. In this case, a defective *DNA-PKcs* gene which is an essential component of the NHEJ repair pathway renders these cells hypersensitive to the lethal effects of ionising radiation and results in a failure of efficient DNA DSB repair. It can be observed that the relative nuclear fluorescence in both the AT5BIVA and XP14BRneo17 cells is almost identical at 24 hrs despite the fact that A-T cells retain more γ-H2AX foci in the nuclei than the XP14BRneo17 cells after 24 hrs. This may be due to differences in the intensity of foci within the nuclei of the cells.

For comparison the total cellular PE fluorescence is also shown in Figure 4. By comparing nuclear and total cellular fluorescence in each cell type, two critical observations are apparent. First, the total level of fluorescence retention each cell line is higher when compared with nuclear only fluorescence. This may be due in part to nonspecific staining of other cellular structures. Second, the difference in fluorescence retention between the cell lines is similar when compared with nuclear-only fluorescence. For example, the repair proficient MRC5-SV1 cells undergo a rapid reduction in fluorescence by 5 hrs indicating efficient repair of DSB, whereas the two DSB repair defective cell lines retain a similarly higher level of fluorescence indicating defective DSB repair.

## Discussion

In this report, we present a quantitative study of radiation-induced γ-H2AX induction in the repair normal (SV40 large T antigen) immortalized human fibroblast cell line MRC5-SV1 and in two SV40 immortalized DNA DSB repair defective fibroblast cell lines XP14BRneo17 and AT5BIVA. To conduct this study, we have used the novel technique of multispectral imaging flow cytometry using the Imagestream^X^. Using this technique, we were able to capture an image of every cell in flow using a maximum of six optical channels. In this case, a bright-field image was collected together with an image of PE staining representing the intensity and number of nuclear γ-H2AX foci and an image was also captured of nuclear location and morphology using Draq 5 staining. Using this method, it was possible to quantitate the frequency and distribution of γ-H2AX foci in a minimum of 20,000 cells for each cell line at each time point which hitherto has been impractical using conventional fluorescence microscopy.

Using imaging flow cytometry, we have been able to detect differences in the induction and repair of DNA DSB (measured by γ-H2AX induction) in the three cell types. For the repair competent MRC5-SV1 cells, there is a rapid and predictable induction of γ-H2AX foci detected at 30 min postirradiation (Fig. 3A) followed by repair of the DSB and loss of γ-H2AX foci. Such a profile of induction and repair is typical of many repair competent cells (*e.g.*, reference 11).

In the AT5BIVA fibroblast cell line derived from a classical ataxia telangiectasia patient, there is again a predictable induction of DNA DSB indicated by the appearance of γ-H2AX foci. However, due to the defect in the *ATM* gene which results in defective DNA DSB repair, there is a persistence of foci in the cells at 24 hrs post 2 Gy gamma irradiation; a result typical of many A-T cells. Interestingly in the DSB repair defective XP14BRneo17 cells, exposure to 2 Gy radiation causes a striking induction of foci to levels consistent with the two other cell types. However, over the course of 24 hrs after irradiation, there is an intermediate or partial retention of γ-H2AX foci in these cells. Previous investigations using this cell line has revealed a splicing defect in the *DNA-PKcs* gene. A functional *DNA-PKcs* gene is central to effective NHEJ repair of DNA DSB (11). This results in a heterozygous phenotype whereby both the normal and a defective splice variant of the *DNA-PKcs* gene are produced. This molecular defect resulted in both clinical and cellular radiation sensitivity (11). In this study of γ-H2AX foci induction, the intermediate retention of foci within the nuclei of these cells after 24 hrs is likely due to the haploinsufficiency of the *DNA-PKcs* gene resulting in partial repair of DNA DSB and retention of ∼ 50% of the foci after 24 hrs.

The profile of γ-H2AX induction in the AT5BIVA cells is markedly different from both the repair normal MRC5-SV1 and repair defective XP14BRneo17 cells. Here, the dramatic induction of foci following exposure to 2 Gy gamma radiation is followed by a retention of the foci for 24 hrs. This is a predictable observation and consistent with the findings of others and due to the well-described defect in the *ATM* gene in these cells results in defective DNA DSB repair (18).

In this study, we additionally conducted a comparison of foci counting using imaging flow cytometry and *in situ* microscopy. While we observed similar profiles of foci induction within the three cell types, it was noticeable that the number of foci scored using *in situ* methods was relatively higher than that observed using the Imagestream^X^ (Figs. 3A and 3B). The principal reason for this difference is likely due to the increased resolution that microscopy affords with the use of a 100× magnification objective. However, it was gratifying to observe that that the relative differences in foci induction between the three cell lines was similar when quantified with both methods. Notwithstanding, without the application of confocal image acquisition there remains user subjectivity in the scoring of foci in DNA damage assays.

We have also been able to compare nuclear fluorescence attributable to specific PE staining with total cellular fluorescence and thus compare fluorescence induction following radiation exposure utilizing imaging flow cytometry (nuclear fluorescence) with zero resolution flow cytometry (total cellular fluorescence). Using either approach, it is possible to detect differences in DNA repair capacity between the repair proficient MRC5-SV1 cells and the two DSB repair defective cell lines AT5BIVA and XP14BRneo17 cells. However, the overall fluorescence detected in whole cells is markedly higher than when fluorescence is calculated from the nuclear region only. The higher relative fluorescence derived from zero resolution flow cytometry may be due to nonspecific staining of other cellular structures.

Overall, the application of multispectral imaging flow cytometry provides a novel and robust mechanism for the estimation and quantitation of γ-H2AX foci in cells. The added ability to visualize each cell in flow with multiple channels provides a quick and accurate method for detecting differences in DNA damage responses in a variety of cell types and the advantage of basing these observations on large cell numbers (many thousands) provides robust data. In addition, the rapid and technically simple method permits high throughput and prompt data analysis.

While the Imagestream^X^ technology at present represents a lower resolution method compared with standard and automated microscopy image capture methods (15–17), the ability to capture images in many thousands of cells in a rapid fashion represents a useful tool for the quantitation of DNA damage and repair in a variety of cell types.

## References

[1] Scott SP, Pandita TK (2006). The cellular control of DNA double strand breaks. J Cell Biochem.

[2] Bourton EC, Plowman PN, Smith D, Arlett CF, Parris CN (2011). Prolonged expression of the γ-H2AX DNA repair biomarker correlates with excess acute and chronic toxicity from radiotherapy treatment. Int J Cancer.

[3] Clingen PH, Wu JY, Miller J, Mistry N, Chin F, Wynne P, Prise KM, Hartley JA (2008). Histone H2AX phosphorylation as a molecular pharmacological marker for DNA interstrand crosslink cancer chemotherapy. Biochem Pharmacol.

[4] Rogakou EP, Pilch DR, Orr AH, Ivanova VS, Bonner WM (1998). DNA double stranded breaks induce histone H2AX phosphorylation on serine 139. J Biol Chem.

[5] Sedelnikova OA, Pilch DR, Redon C, Bonner WM (2003). Histone H2AX in DNA damage and repair. Cancer Biol Ther.

[6] Burma S, Chen BP, Chen DJ (2006). Role of non-homologous end joining (NHEJ) in maintaining genomic integrity. DNA Repair (Amst).

[7] Bassing CH, Alt FW (2004). The cellular response to general and programmed DNA double strand breaks. DNA Repair (Amst).

[8] Riballo E, Critchlow SE, Teo SH, Doherty AJ, Priestley A, Broughton B, Kysela B, Beamish H, Plowman N, Arlett CF, Lehmann AR, Jackson SP, Jeggo PA (1999). Identification of a defect in DNA ligase IV in a radiosensitive leukaemia patient. Curr Biol.

[9] Musio A, Marrella V, Sobacchi C, Rucci F, Fariselli L, Giliani S, Lanzi G, Notarangelo LD, Delia D, Colombo R, Vezzoni P, Villa A (2005). Damaging-agent sensitivity of Artemis-deficient cell lines. Eur J Immunol.

[10] Friesner JD, Liu B, Culligan K, Britt AB (2005). Ionizing radiation-dependent gamma-H2AX focus formation requires ataxia telangiectasia mutated and ataxia telangiectasia mutated and Rad3-related. Mol Biol Cell.

[11] Abbaszadeh F, Clingen PH, Arlett CF, Plowman PN, Bourton EC, Themis M, Makarov EM, Newbold RF, Green MHL, Parris CN (2010). A novel splice variant of the DNA-PKcs gene is associated with clinical and cellular radiosensitivity in a xeroderma pigmentosum patient. J Med Genet.

[12] Werbrouck J, De RE, Beels L, Vral A, Van EM, De NW, Thierens H (2010). Prediction of late normal tissue toxicity complications in RT treated gynaecological cancer patients: Potential of the gamma-H2AX foci assay and association with chromosomal radiosensitivity. Oncol Rep.

[13] Macphail SH, Banath JP, Yu TY, Chu EHM, Lambur H, Olive PL (2003). Expression of phosphorylated histone H2AX in cultured cell lines following exposure to X-rays. Int J Radiat Biol.

[14] Mistrik M, Oplustilova L, Lukas J, Bartek J (2009). Low-dose DNA damage and replication stress responses quantified by optimised automated single-cell image analysis. Cell Cycle.

[15] Furuta T, Hayward RI, Meng L-H, Takemura H, Aune GJ, Bonner WM, Aladjem MI, Kohn KW, Pommier Y (2006). p21^CDKN1A^ allows the repair of replication-mediated DNA double-strand breaks induced by topoisomerase I and is activated by the checkpoint kinase inhibitor 7-hydroxystaurosporine. Oncogene.

[16] Schmid TE, Dollinger G, Beisker W, Hable V, Greubel C, Auer S, Mittag A, Tarnok A, Friedl AA, Molls M, Roper B (2010). Differences in the kinetics of gamma-H2AX fluorescence decay after exposure to low and high LET radiation. Int J Radiat Biol.

[17] Zhao H, Traganos F, Darzynkiewicz Z (2010). Kinetics of the UV-induced DNA damage response in relation to cell cycle phase. Correlation with DNA replication. Cytometry Part A.

[18] Murnane JP, Fuller LF, Painter RB (1985). Establishment and characterization of a permanent pSV ori-transformed ataxia-telangiectasia cell line. Exp Cell Res.

[19] Arlett CF, Green MHL, Priestley A, Harcourt SA, Mayne LV (1988). Comparative human cellular radiosensitivity. I. The effect of SV40 immortalisation on the gamma-radiation survival of skin derived fibroblasts from normal individuals and from ataxia telangiectasia patients and heterozygotes. Int J Radiat Biol.

[20] Filby A, Perucha E, Summers H, Rees P, Chana P, Heck S, Lord GM, Davies D (2011). An imaging flow cytometric method for measuring cell division history and molecular symmetry during mitosis. Cytometry Part A.

