# Supplementary material for: Multispectral imaging flow cytometry reveals distinct frequencies of γ-H2AX foci induction in DNA double strand break repair defective human cell lines
Source: Cytometry A. 2012 Feb;81A(2):130–7. doi: 10.1002/cyto.a.21171 (PMC3489045; doi:10.1002/cyto.a.21171)
Supplement: Supplementary file 1 [file cyto0081A-0130-SD1.doc]

**Supplementary Figure 1**

|  | Ch01 | Ch02 | Ch03 | Ch04 | Ch05 | Ch06 |
| --- | --- | --- | --- | --- | --- | --- |
| Ch01 | 1 | 0 | 0.061 | 0 | 0.036 | 0 |
| Ch02 | 0 | 1 | 0.277 | 0 | 0.125 | 0 |
| Ch03 | 0 | 0 | 1 | 0 | 0.100 | 0 |
| Ch04 | 0 | 0 | 0.518 | 1 | 0.143 | 0 |
| Ch05 | 0 | 0 | 0.208 | 0 | 1 | 0 |
| Ch06 | 0.02 | 0 | 0.087 | 0 | 0.352 | 1 |

Supplementary Figure 2 shows an example of a typical compensation matrix derived from AT5BIVA cells following exposure to 2 Gy gamma radiation and fixed and stained at 30 minutes post irradiation. Positive channels are channel 3 (PE staining for γ-H2AX) and channel 5 (Draq 5 staining for DNA in the nucleus). Coefficients on each channel are largest in those channels juxtaposed to channels 3 and 5 but decline in subsequent channels representing reduced leakage of fluorescence signal into these channels.
