# Supplementary material for: Multispectral imaging flow cytometry reveals distinct frequencies of γ-H2AX foci induction in DNA double strand break repair defective human cell lines
Source: Cytometry A. 2012 Feb;81A(2):130–7. doi: 10.1002/cyto.a.21171 (PMC3489045; doi:10.1002/cyto.a.21171)
Supplement: Supplementary file 2 [file cyto0081A-0130-SD2.doc]

**Cytometry Part A**

**Author Checklist: Location of MIFlowCyt-Compliant Items**

| **Requirement** | **Requested Information or Specific Location in Manuscript** |
| --- | --- |
|  |  |
| 1.1. Purpose | Abstract: Page 2 |
| 1.2. Keywords | Page 2 |
| 1.3. Experiment variables | Not applicable |
| 1.4. Organization name and address | Page 1: Title page |
| 1.5. Primary contact name and email address | Page 1: Title page |
| 1.6. Date or time period of experiment | Materials and Methods: Page 13 |
| 1.7. Conclusions | Discussion: Page 18-20 |
| 1.8. Quality control measures | Not applicable |
| 2.1.1.1. (2.1.2.1., 2.1.3.1.) Sample description | Materials and Methods: Page 7 |
| 2.1.1.2. Biological sample source description | Materials and Methods: Page 7 |
| 2.1.1.3. Biological sample source organism description | Materials and Methods: Page 7 |
| 2.1.2.2. Environmental sample location | Not applicable |
| 2.3. Sample treatment description | Materials and Methods: Page 7 |
| 2.4. Fluorescence reagent(s) description | Materials and Methods: Page 8 |
| 3.1. Instrument manufacturer | Materials and Methods: Page 9 |
| 3.2. Instrument model | Materials and Methods: Page 9 |
| 3.3. Instrument configuration and settings | Materials and Methods: Page 9 |
| 4.1. List-mode data files | List mode data files are not generated with Imagestream. Sample data analysis files which are of very large size will not upload through Scholar One or transmit via email. Data files will be provided if necessary if the paper is accepted for publication. All data files are kept on the Brunel University network |
| 4.2. Compensation description | Materials and Methods: Page 10 |
| 4.3. Data transformation details | Not applicable |
| 4.4.1. Gate description | Materials and Methods: Page 11 and Supplementary Figures 2A-C |
| 4.4.2. Gate statistics | Materials and Methods: Page 11 and Supplementary Figures 2A-C |
| 4.4.3. Gate boundaries | Materials and Methods: Page 11 and Supplementary Figures 2A-C |

**Notes**

Feel free to use more space than allocated.

You can embed graphics/figures in this document, if needed.

Please make sure to save the document in Microsoft Word version 2003 or older, before uploading to ScholarOne Manuscripts.

For any questions, please contact the Cytometry Part A editorial office at [Cytometrya@wiley.com](mailto:Cytometrya@wiley.com).
