# Supplementary figures and images for: Multispectral imaging flow cytometry reveals distinct frequencies of γ-H2AX foci induction in DNA double strand break repair defective human cell lines
Source: Cytometry A. 2012 Feb;81A(2):130–7. doi: 10.1002/cyto.a.21171 (PMC3489045; doi:10.1002/cyto.a.21171)

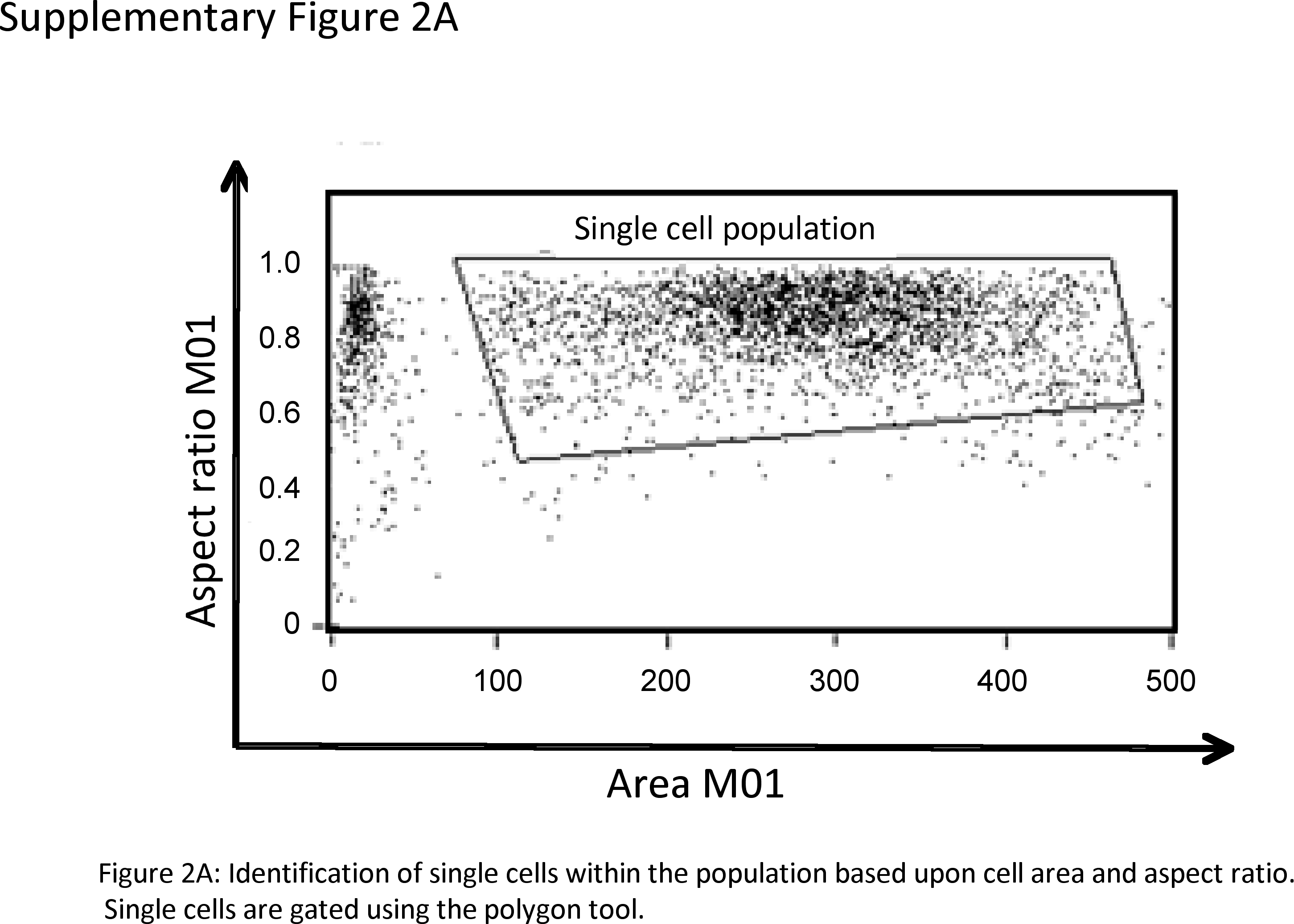

Supplement: Supplementary file 3 [file cyto0081A-0130-SD3.tif]

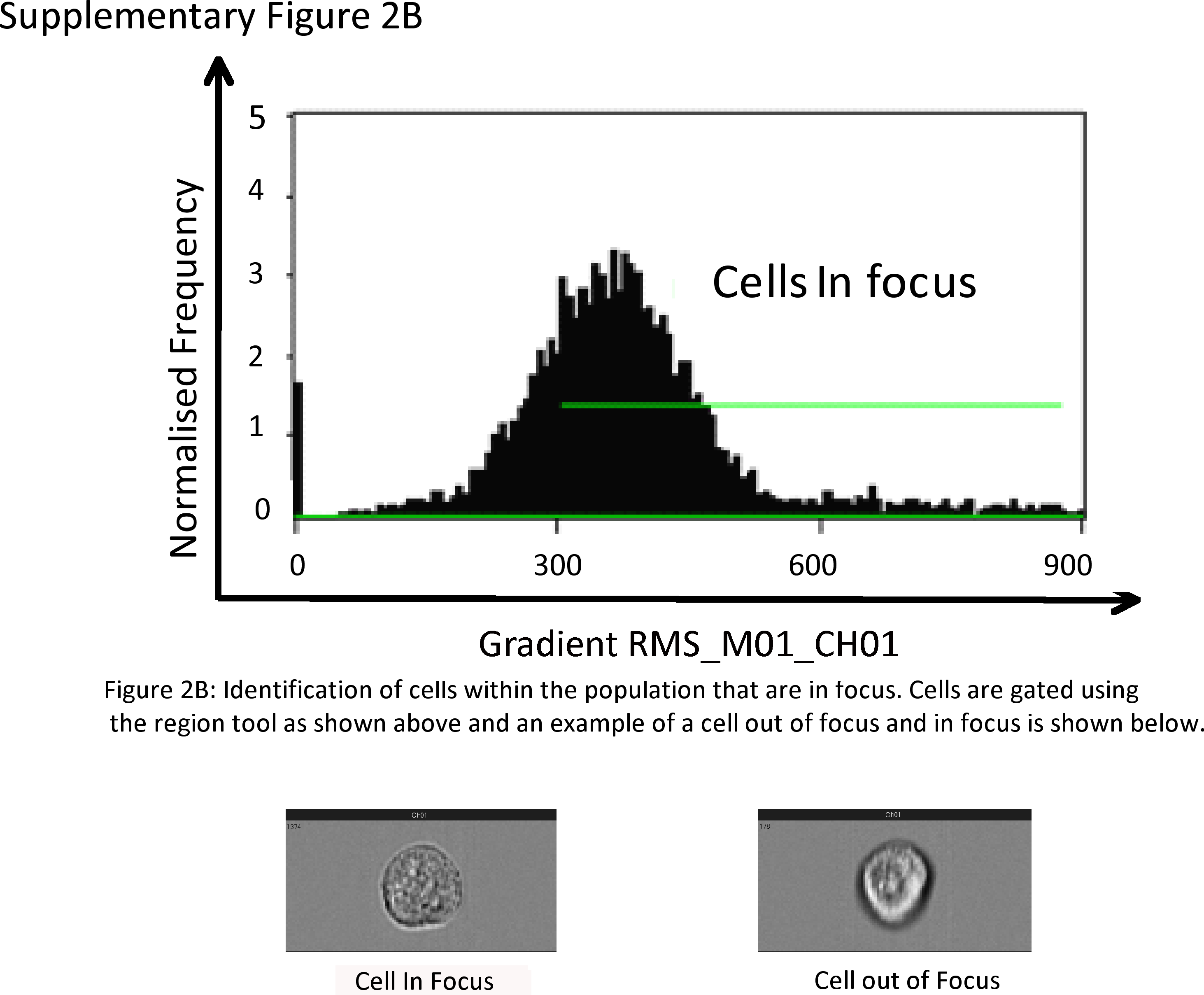

Supplement: Supplementary file 4 [file cyto0081A-0130-SD4.tif]

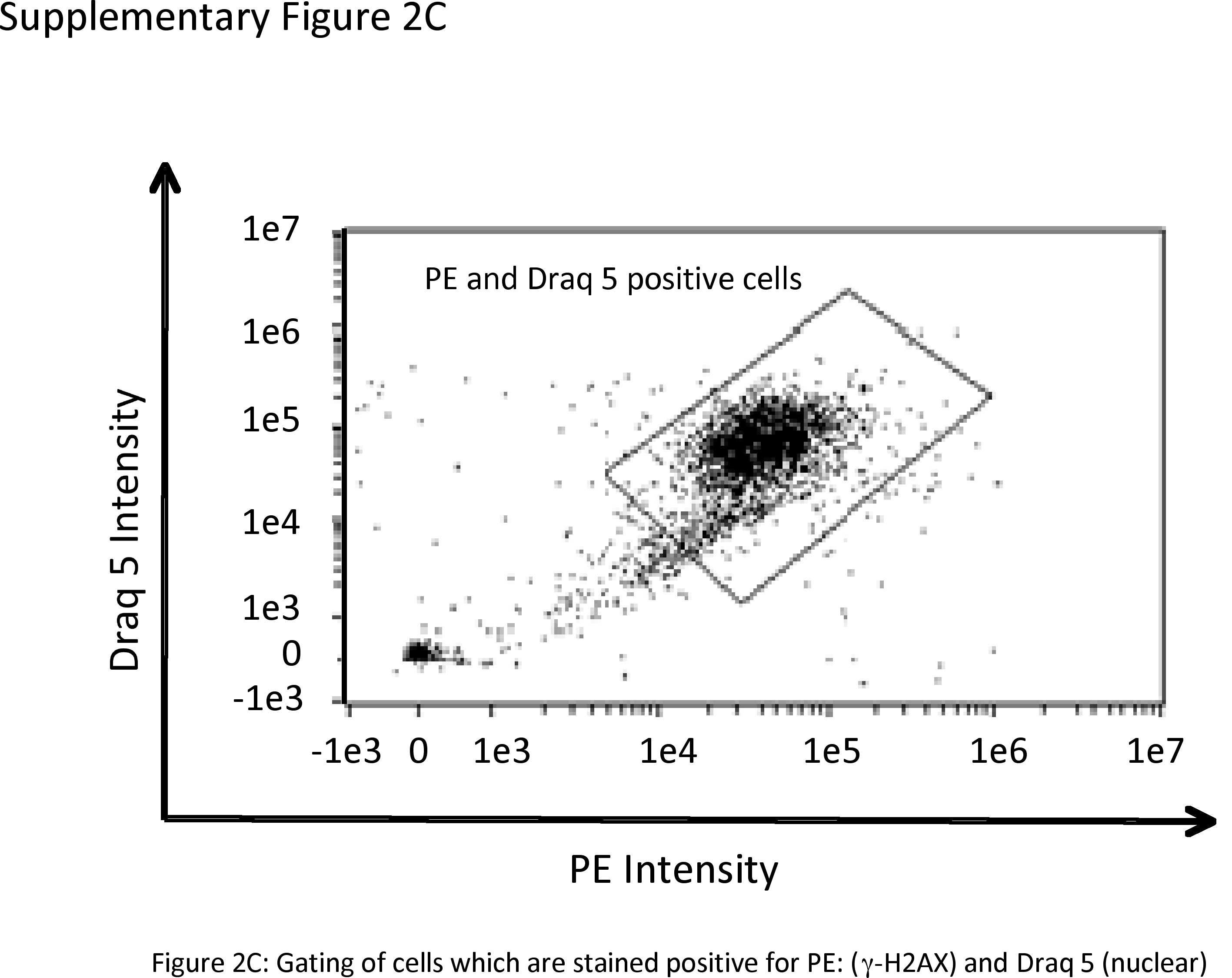

Supplement: Supplementary file 5 [file cyto0081A-0130-SD5.tif]

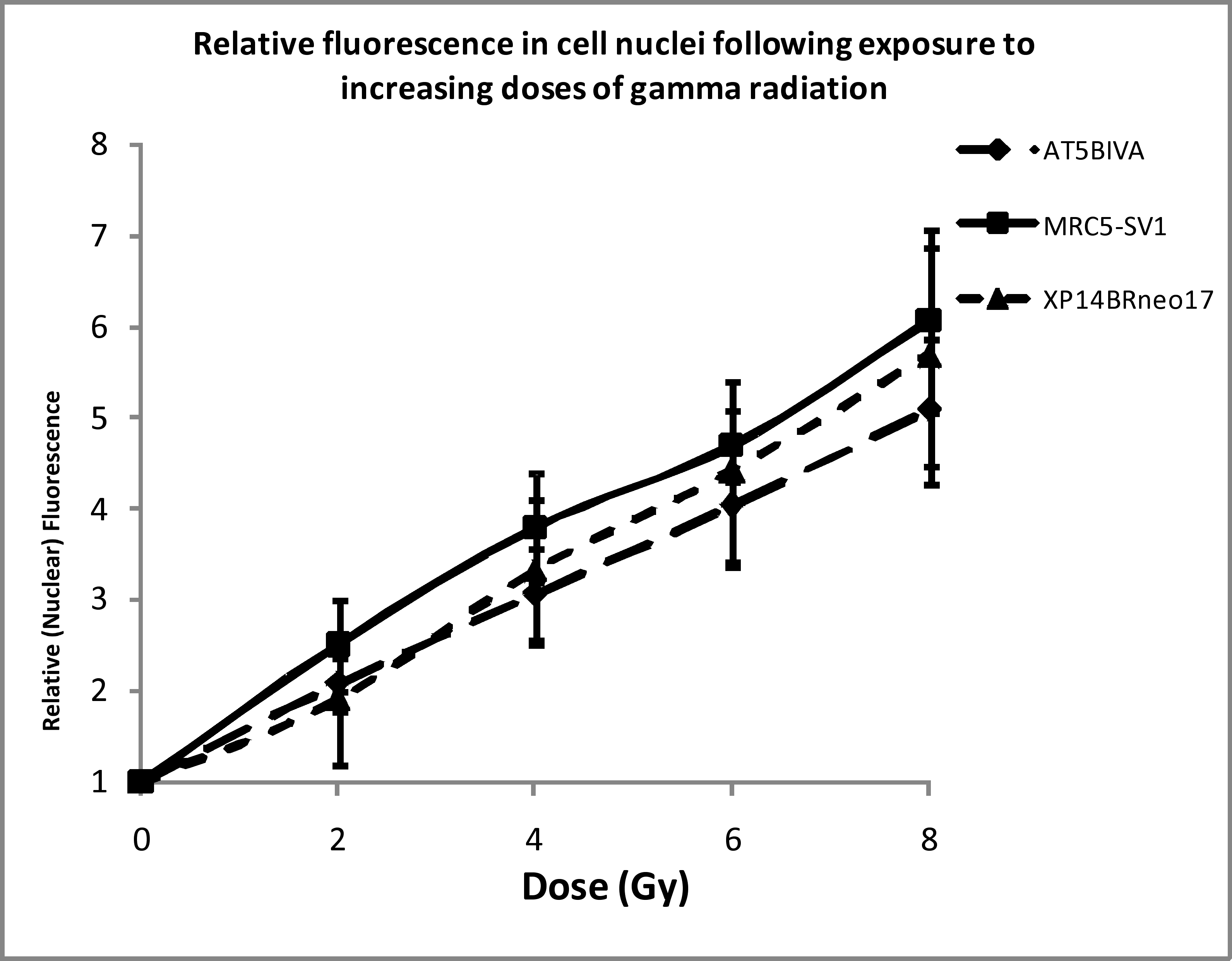

Supplement: Supplementary file 6 [file cyto0081A-0130-SD6.tif]
